# Neuroprotective Effect of Total and Sequential Extract of *Scrophularia striata Boiss*. in Rat Cerebellar Granule Neurons Following Glutamate- Induced Neurotoxicity: An *In-vitro *Study

**Published:** 2013

**Authors:** Parvin Salavati, Mina Ramezani, Hamid R Monsef-Esfahani, Reza Hajiagha, Maliheh Parsa, Shoreh Tavajohi, Seyed Nasser Ostad

**Affiliations:** a *Department of Animal Science, Faculty of Biology, University of Payam Noor, Tehran, Iran. *; b *Department of Biology, Faculty of Basic Sciences, Islamic Azad University, Ashtian, Iran. *; c *Department of Pharmacognosy, Faculty of Pharmacy, Tehran University of Medical Sciences, Tehran, Iran.*; d *Department of Pharmacognosy and Pharmacy of Medicinal Plant Research Center, Institute of Medicinal Plants, ACECR, Karaj, Iran. *; e *Department of Toxicology and Pharmacology, Faculty of Pharmacy, Tehran University of Medical Sciences, Tehran, Iran.*

**Keywords:** CGNs, Glutamate Neurotoxicity, *Scrophularia striata*, Neuroprotection

## Abstract

Neuroprotective effect of the extract from aerial parts of *Scrophularia striata Boiss *(*Scrophulariaceae*) was investigated against glutamate-induced neurotoxicity on cultured rat pups Cerebellar Granule Neurons (CGNs). CGNs from 8 days old Sprague-Dawley rat were prepared and cultured. The experiments were performed after 8 days in culture. The plant was collected from the northeastern part (Ruin region) of Iran and air-dried at room temperature. The total extract was prepared with maceration of prepared powder in ethanol 80% for three times.

Sequential extracts were obtained using dried and powdered aerial parts with increasingly polar solvents: petroleum ether, chloroform, ethyl acetate and methanol 80% solution. Cultured cells were exposed to 125 μM of glutamate for 12 h following a 24 h of incubation with test fractions at concentration of 10 mcg/mL. Morphological assay was performed using invert light microscope after fixation and staining with haematoxylin. Neuronal viability was measured using MTT assay. Statistical analysis was done using SPSS software. One way analysis of variance (ANOVA) was performed by Tukey post-hoc test. Values were considered statistically significant when p-value ≤ 0.05. Results of this study showed a significant neuroprotective activity of high polarity methanolic fraction of aerial parts of *Scrophularia striata *against glutamate-induced neurotoxicity in a dosedependent manner. Treatment with 10 mcg/mL of the fractions showed the best result.

## Introduction

Glutamate, the major excitatory neurotransmitter in CNS involved in fast synaptic transmission, neuronal plasticity, outgrowth and survival, memory, learning and behavior (1). However, it can be a potential neurotoxic substance at concentrations higher than normal level, resulting in neuron damage or death (2). Glutamate-mediated neurotoxicity appears to play a crucial role in several neuropathological disorders, particularly in Alzheimer’s disease, Parkinson’s disease, epilepsy and ischemic stroke (3). The most relevant biochemical events in glutamate-mediated neurotoxicity might be the acute influx of excess Ca^2+ ^sequentially followed by over activation of Ca^2+^-dependent enzymes, such as nitric oxide synthesis (NOS), increased formation of reactive oxygen species (ROS) and lipid per oxidation (4, 5).

Many species of the genus *Scrophularia *(*Scrophulariaceae*) have been used traditionally as a remedy to cure illnesses such as scabies, tumors, eczema, psoriasis, inflammatory disorders fever, constipation, pharyngitis, neuritis and laryngitis affections (6-9).

 Neuroprotective effects of some plants from this genus have been also considered in traditional medicine of Iran as well as some other countries such as South Korea (10). Neuroprotective (11) Cognitive-enhancing (10) and Anti-amnesic (12) properties of *Scrophularia buergeriana *have been reported previously. To date, many active components or crude extracts prepared from *Scrophularia buergeriana *roots have been reported to be neuroprotective potential* in-vitro *against glutamate excitotoxicity (11, 13). *Scrophularia striata Boiss*, another plant from this genus has been reported to have some of pharmacological effects such as Analgesic (14), Anti-microbial (15), nephroprotective (16) and nitric oxide suppressive (17) properties. Although this plant is commonly used in Iranian folk medicine for its neuroprotective effect, up to now, no study has been performed to investigate this effect using *in-vitro*/*in-vivo *biological instruments. Rat cerebellar granule neurons (CGNs) are the most common instrument in study of oxidative stress, excitotoxicity and preconditioning. In 8 days old rat neonates, CGNs are still developing, but have begun to express NMDA receptors (18). Culture of CGNs is well recognized as an effective tool for investigation in excitotoxicity (19).

In an attempt to find natural products with neuroprotective activity as a natural remedy against glutamate-mediated neurological disorders, we designed this study to evaluate the neuroprotective activity of total and sequential extracts from the aerial parts of *Scrophularia* *striata Boiss *(*Scrophulariaceae*) on primary cultures of rat CGNs after exposure to the glutamate. To the best of our knowledge, this is the first evaluation of neuroprotective effect of this plant and the results of this study may help to find a useful and natural treatment to cure glutamate exitotoxicity-related disorders.

## Experimental

*Animals*

Wister albino rats, weighing 200-250 g, were obtained from Pasteur Institute, Tehran, Iran. Animal experiments were conducted in accordance with current ethical regulations on animal research in Tehran University of Medical Sciences. Animals were randomized and housed in five plastic-based cages (40 × 26 × 15 cm^3^), each containing one male and two females, and were maintained under standard laboratory conditions (temperature of 20 ± 2°C, relative humidity of 40-45% and light-dark cycle of 12:12 h). Standard rat chow and tap water were available ad libitum.

*Cell culture*

**Figure 1:**
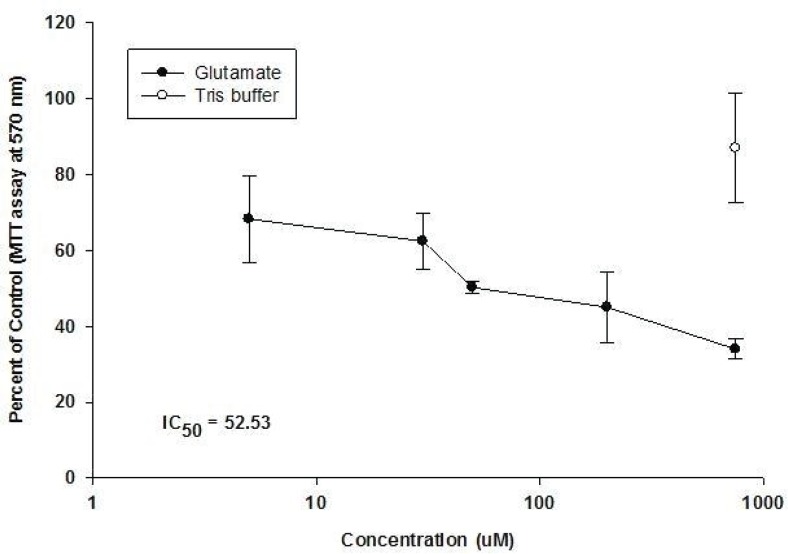
Cerebellar granule neuron cultures of 8-day rat neonate at 8DIV were stained with hematoxylin, Original magnifications 200× (A), and 400× (B).

Rat cerebellar granule neurons were prepared from 8 days old Sprague-Dawley rat as described previously (20). Briefly, neurons were dissociated from freshly dissected cerebellum by mechanical disruption in presence of Trypsin/ EDTA and 2400 U/mL DNase I and seeded into 0.1 mg/mL poly-D-lysine-coated hydrobromide in 24-well plastic plates at density of 1.25 × 10^6 ^Cells/mL in Basal modified Eagle’s medium supplemented with 10% fetal bovine serum (FBS), 25 mM KCl, 2 mM L-Glutamine and Gentamicin 0.1 mg/mL, and were incubated at 37°C in a humidified atmosphere of 95% air and 5% CO_2_. The replication of non-neuronal cells was prevented by adding 10 μM cytosine arabinoside 18-24 h after plating. Using this protocol, 95-99% of cultured cells were granule neurons (Figure 1). The experiments were performed after 8 days in culture.

*Plant collection*

The aerial parts of *Scrophularia striata Boiss *were collected from the northeastern part of Iran, in Ruin region. A sample was authenticated by Dr. F. Attar and a voucher specimen was preserved in the Faculty of Sciences’ Herbarium, Tehran University of Medical Sciences, Tehran, Iran (TUH No: 36501). 

*Total extract*

Aerial parts of the plant were air-dried, powdered (100 g) and macerated with an 80% ethanol solution for 3 days with three changes of the solution. The resulting extract was filtered and evaporated under vacuum into a dried powder (10.3 g, 10.3%).

*Sequential extracts*

Solvents were from Merck Company. Different extracts were sequentially prepared using 500 g dried and powdered aerial parts of the plant with serial increase in polarity of solvents: petroleum ether (A, 3.3 g dry weight or responding to 0.6%) (bp.70-100°C), chloroform (B, 5.7 g dry weight corresponding to 1.1%), ethyl acetate (C, 6.4g dry weight corresponding to 1.2%) and 80% methanol solution (D, 8.2 dry weight corresponding to 1.6%). The extracts were dried under vacuum and dissolved in dimethyl sulfoxide (DMSO). The final concentration of DMSO was 0.1% (v/v) which was not toxic on the rat cortical neurons.

*Morphological assay*

For the light microscopy examinations, the cultures were washed with PBS and fixed with 10% formaldehyde for 5 min. Staining was performed using hematoxylin after being washed with PBS. Cells were washed again two times with PBS and were observed using an invert light microscope (Leitz Germany).

*Determination of glutamate IC*_50_

Selected cultures were exposed to a wide range of glutamate (5, 30, 50, 200, 500, 750 μM) for 12 h at room temperature in a standard Tris-buffered salt solution to calculate the IC_50_. Concentration of 125 μM was selected and used to continue the research.

*Neuroprotection assay*

All tested compounds were dissolved in DMSO and added to cell culture medium to bear the final concentrations of 0.1, 10, 25, 50, 75, 100 μg/mL. The final culture concentration of DMSO was 0.1% v/v which was not toxic for the cells. Neuronal viability was determined using MTT assay and IC_50_ was calculated. Concentration of 10 μM of compounds was selected to continue the project. Cultured CGNs were exposed to the medium containing the selected concentration of test compounds (10 μg/mL) for 1 h followed by exposure to the concentration of 125 μM of glutamate for 12 h. After washing, incubation was continued at concentration of 10 μg/mL of test fractions for 24 h. Neuronal viability was determined using MTT assay at 570 nm (690 nm reference wavelength).

*Statistical analysis*

Three independent experiments in triplicate were done for all assays (21). All data were expressed as mean ± SD of number of experiments. Statistical analysis was performed with one way analysis of variance (one way ANOVA) after confirmation of normal distribution, and group means were compared by post-hoc test of Tukey multiple comparisons of means. Values were considered statistically significant when p-value ≤ 0.05. Calculations of IC_50_ were made using the Microsoft office Excel and Sigma Plot version 11.0 (Systat Software, Inc.). 

## Results

**Figure 2:**
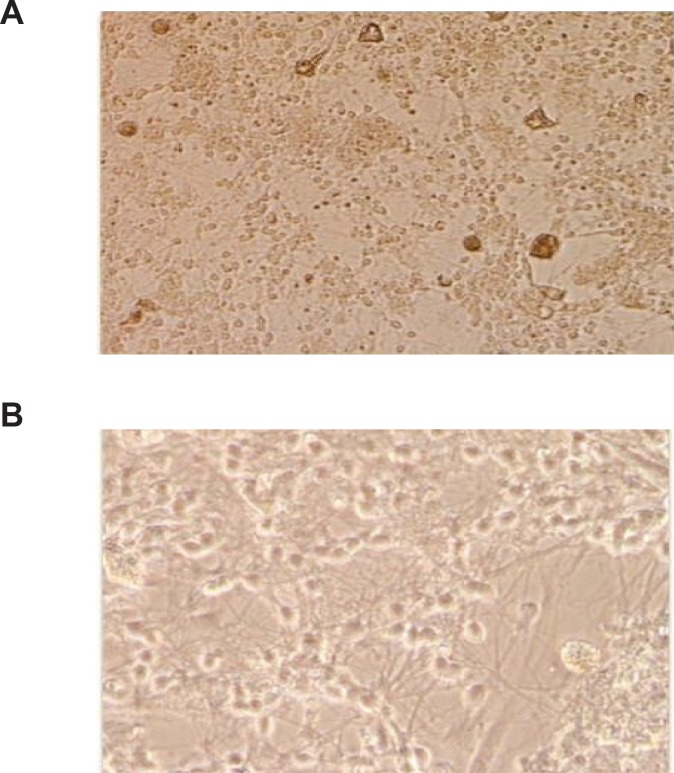
Effect of Glutamate added in Tris buffer for 12h at 8 DIV on CGNs at 8 day old rat neonate (Means ± S.D, n = 3, p-value > 0.05).

Our results showed that glutamate has a cytotoxic effect on CGNs (Figure 2). 

The application of glutamate, at concentrations over 5 μM for 12 h at 8 DIV caused a dosedependent reduction in neuronal viability up to a concentration of 750 μM, beyond which no further damage due to glutamate-mediated toxicity was observed (IC_50_ = 52.53). Cultured CGNs showed that the rounded cell bodies and phase-bright were severely damaged. Immediately after the brief exposure to glutamate, phase-contrast microscopy showed an increased darkness and granularity of cell bodies and by 24 h, most of the neurons had disappeared. It was noticeable that a relatively sharp drop in viability compared with the sham control was noted when the normally non-toxic concentration of 5 μM glutamate was applied, suggesting that the ‘sham control’ procedure may not have accounted for all of the damages inflicted by the treatment procedure. The presence of glutamate in Tris buffer causes a larger rise due to the release of endogenous neuronal glutamate and decreased of cell viability. 

The IC_50_ for neuroprotective effects of the extracts were calculated as the doses at which 50% neuroprotective effects occurred compared to untreated control cells. Calculated IC_50_s for total and sequential fractions showed that none of the fractions showed cytotoxic effects on cultured CGNs at selected concentration for neuroprotective studies (Table 1). 

**Table 1:** Calculated IC_50_ for total and sequential fractions based on the data from cytotoxic assays.

**Extract**	**IC**_50_ ** (mcg/mL)**
Total extract	30
Chloroform fraction	> 100
Methanolic fraction	> 100
Ethyle acetate fraction	> 100
Petroleum	> 100

**Figure 3:**
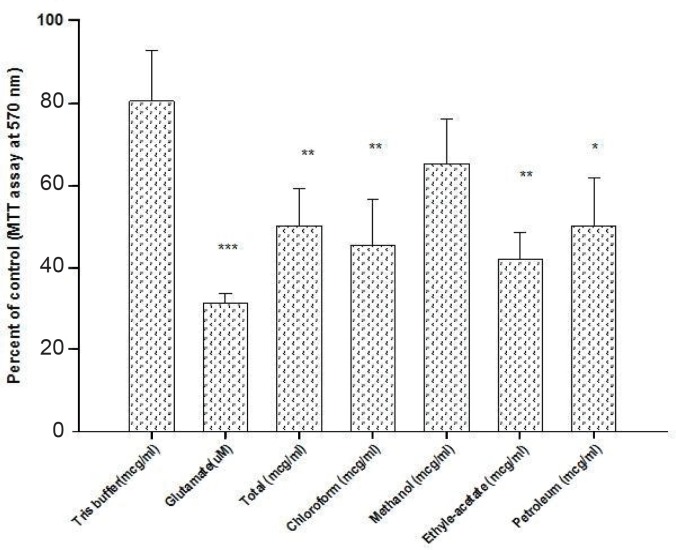
Neuroprotective effect of *Scrophularia striata *on the glutamate- induced neurotoxicity at 8 DIV on CGNs at 8 day old rat neonate (Means ± SD, n = 3, p-value > 0.05).

All fractions showed a marginal inhibition of glutamate-induced neurotoxicity (Figure 3). As shown, of the five different fractions of *Scrophularia striata Boiss*, MeOH fraction was observed to have the most neuroprotective effect against glutamate-induced neurotoxicity in a dose-dependent manner.

## Discussion

*Scrophularia striata *(*Scrophulariaceae*) is used traditionally as a medicinal herb in Iran and some other countries such as South Korea and china. Recently, some novel pharmacological actions of *Scrophularia striata *were discovered, such as antimicrobial effects (14), inhibitory effect on matrix metalloproteinase (MMPs) (22), suppression of NO production in activated murine peritoneal macrophages *ex-vivo *(16) and stimulatory effects on human fibroblast cells proliferation and on improvement of wound healing and anti-tumor activity (23). According to the literature, several *Scrophularia *species such as *Scrophularia buergeriana *have been investigated and found to contain some classes of secondary metabolites including iridous, phenylpropanoids, phenolic acids, flavonoid and saponins. Some of these compounds were shown to have different pharmacological and therapeutic effects including neuroprotective properties (11, 24). However, no study has been performed yet to investigate the neuroprotective effects of *S. striata*.

The initial purpose of the present study was to investigate the neuroprotective effect of *Scrophularia striata Boiss *extract against glutamate-induced neuronal injury in primary cultured rat CGNs. We observed a significant protective effect of MeOH fraction against glutamate neurotoxicity, although total EtOH extract and other studied fractions have also attenuated the glutamate neurotoxicity. Based on the literature, five known compounds, including cinnamic acid, three flavonoids (quercetin, isorhamnetin-3-O-rutinoside and nepitrin) and one phenylpropanoid glycoside (acteoside 1) were previously isolated from the extract of the aerial parts of *S. striata *from 80% methanolic fraction (25). Cinnamic acid and related compounds have been reported to attenuate glutamate-induced neurotoxicity. Flavonoids found in this plant have been showed to reduce oxidative stress which is one of the established mechanisms of glutamate-induced neurotoxicity. Since the best neuroprotective results were dominantly observed from methanolic fraction which contains these neuroprotective compounds, it could be possible that at least some part (of course not completely) of observed neuroprotective effect is a result of these compounds. However, more studies should be designed to find other compounds involved in this effect since the presented data about isolated compounds from the plat *S. striata *are not enough and this study is the first evaluation of neuroprotective properties of this plant. Detailed mechanism of this observed effect also remains as an open question and screening of genes and proteins whose expression levels are modified by long-term treatment with methanolic fraction may provide useful information concerning the molecular targets mediating neuroprotection. 

Taken together, the extract from aerial parts of *Scrophularia striata Boiss *has neuroprotective effect and MeOH fraction showed the best result on CGNs against glutamate-induced neurotoxicity. 
